# Improvement of plant resistance to geminiviruses via protein de-S-acylation

**DOI:** 10.1007/s44154-024-00166-w

**Published:** 2024-04-25

**Authors:** Yawen Zhao, Zhenggang Li, Zhiying Wang, Liting Huang, Gongda Li, Xiaoshi Liu, Meiqi Yuan, Wei Huang, Lishan Ling, Chengwei Yang, Zifu He, Jianbin Lai

**Affiliations:** 1 Guangdong Provincial Key Laboratory of Biotechnology for Plant Development, School of Life Science, South China Normal University, Guangzhou, 510631 China; 2 Guangdong Provincial Key Laboratory of High Technology for Plant Protection, Plant Protection Research Institute, Guangdong Academy of Agricultural Sciences, Guangzhou, 510640 China

**Keywords:** Geminivirus, Plant resistance, Protein de-S-acylation, Protein S-acylation

## Abstract

Geminiviruses are an important group of viruses that infect a variety of plants and result in heavy agricultural losses worldwide. The homologs of C4 (or L4) in monopartite geminiviruses and AC4 (or AL4) in bipartite geminiviruses are critical viral proteins. The C4 proteins from several geminiviruses are the substrates of S-acylation, a dynamic post-translational modification, for the maintenance of their membrane localization and function in virus infection. Here we initiated a screening and identified a plant protein ABAPT3 (Alpha/Beta Hydrolase Domain-containing Protein 17-like Acyl Protein Thioesterase 3) as the de-S-acylation enzyme of C4 encoded by BSCTV (Beet severe curly top virus). Overexpression of ABAPT3 reduced the S-acylation of BSCTV C4, disrupted its plasma membrane localization, inhibited its function in pathogenesis, and suppressed BSCTV infection. Because the S-acylation motifs are conserved among C4 from different geminiviruses, we tested the effect of ABAPT3 on the C4 protein of ToLCGdV (Tomato leaf curl Guangdong virus) from another geminivirus genus. Consistently, ABAPT3 overexpression also disrupted the S-acylation, subcellular localization, and function of ToLCGdV C4, and inhibited ToLCGdV infection. In summary, we provided a new approach to globally improve the resistance to different types of geminiviruses in plants via de-S-acylation of the viral C4 proteins and it can be extendedly used for suppression of geminivirus infection in crops.

**Supplementary Information:**

The online version contains supplementary material available at 10.1007/s44154-024-00166-w.

## Introduction

Geminiviruses are a group of single-stranded DNA viruses that infect a variety of plants and result in heavy agricultural losses worldwide, thus it is important to improve resistance to geminiviruses in plant species (Cana-Quijada et al. 2020). After infection mediated by insects, geminiviruses amplify and spread in plant tissues via the interplay between viral factors and host components, resulting in serious systemic symptoms (Gutierrez 2000). The homologs of C4 (or L4) in monopartite geminiviruses and AC4 (or AL4) in bipartite geminiviruses are critical viral proteins, that interact with host factors and enhance symptom development, suppress gene silencing, or mediate virus movement (Kumar and Dasgupta 2023; Medina-Puche et al. 2021). Geminivirus C4 functions via interaction with plant components, such as kinases, and C4 proteins are also targets of host machines (Mei et al. 2020; Zeng et al. 2018). Previous studies showed that C4 homologs from different geminiviruses, including BSCTV (Beet severe curly top virus) and MYMV (Mungbean yellow mosaic virus), are S-acylated in plant cells for maintenance of their membrane localization and function in virus infection (Carluccio et al. 2018; Li et al. 2018).

S-acylation (also named S-palmitoylation) is a dynamic post-translational lipidation, which transfers long-chain fatty acids such as palmitates to the cysteine residues of proteins to increase the membrane affinity of target proteins. S-acylation is a reversible modification mediated by protein acyltransferases and de-S-acylation enzymes (Linder and Deschenes 2007). Interestingly, S-acylation occurs on proteins encoded by viruses of both animal and plant hosts (Veit 2012), but suppression of virus infection by de-S-acylation enzymes has not yet been reported in any species. Recently, we characterized a group of protein de-S-acylation enzymes with 11 members in Arabidopsis, named Alpha/Beta Hydrolase Domain-containing Protein 17-like acyl protein thioesterases (ABAPTs) (Liu et al. 2021). Therefore, here we performed a screening to identify the de-S-acylation enzyme of C4 for the improvement of plant resistance to geminiviruses.

## Results and discussion

**Fig. 1:**
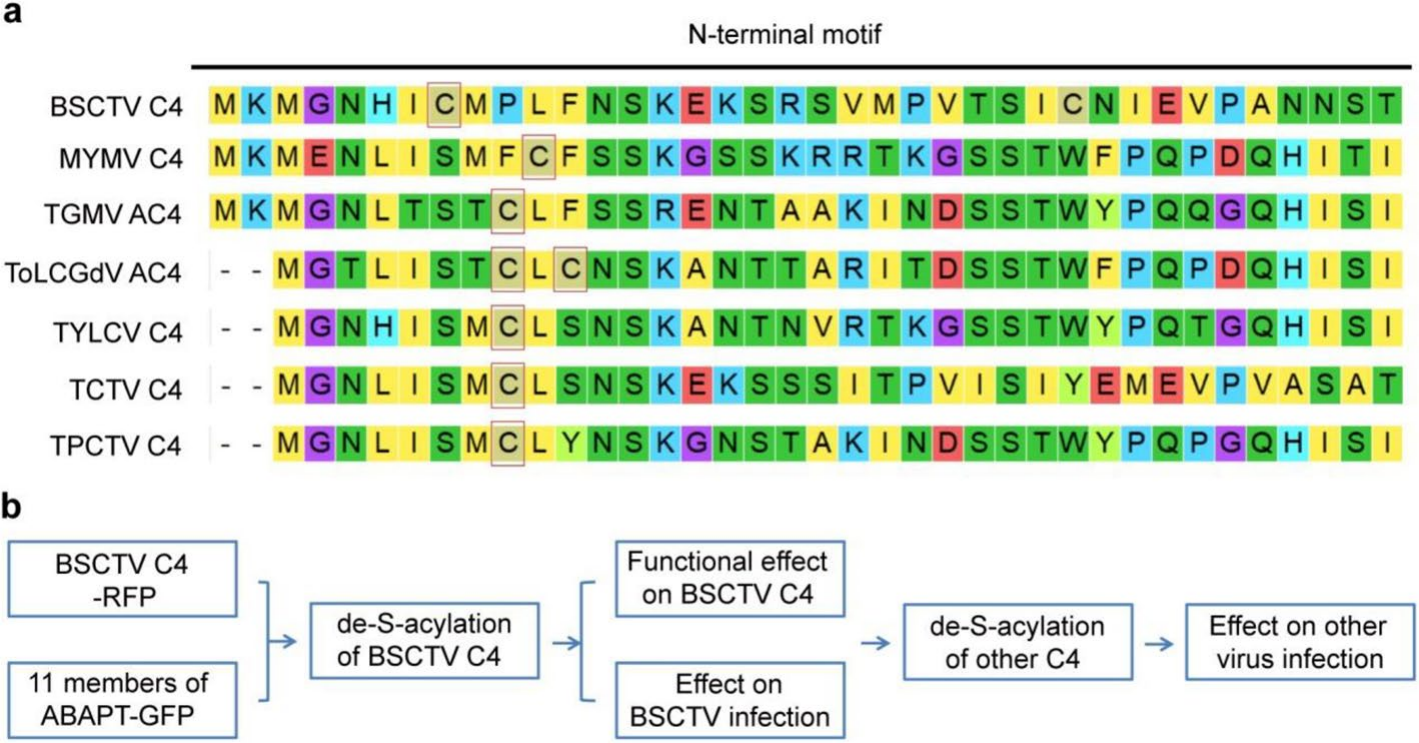
The S-acylation motifs in different C4 proteins and the strategy for geminivirus resistance via C4 de-S-acylation. **a** Sequence alignment of C4 or AC4 proteins from different geminiviruses. Full-length proteins were used for analysis via ClustalW, and the alignments of N-terminal regions are shown. Conserved residues are shown with the ClustalW color code. The S-acylation sites predicted by CSS-PALM are indicated by red boxes. **b** The strategy for improving geminivirus resistance by de-S-acylation of C4 proteins. First, identification of the de-S-acylation enzyme of BSCTV C4 via co-overexpression of C4 distinctly with 11 ABAPT members; second, determination of the effect of overexpression of the identified enzyme on BSCTV C4 and BSCTV infection; third, detection of the effect of the identified enzyme on function of other C4 and infection of other geminiviruses

To establish a method for the suppression of geminivirus infection via protein de-S-acylation, it is important to check the conservation of this modification on C4 proteins. Because a potential S-acylation motif may exist in different geminivirus C4 proteins (Medina-Puche et al. 2021), we aligned BSCTV C4 (Lai et al. 2009) with several other well-studied C4/AC4 proteins from different geminiviruses, including MYMV (Carluccio et al. 2018), TGMV (Tomato golden mosaic virus) (Pooma and Petty 1996), TYLCV (Tomato yellow leaf curl virus) (Jupin et al. 1994), ToLCGdV (Tomato leaf curl Guangdong virus) (Li et al. 2020), TCTV (Turnip curly top virus), and TPCTV (Tomato pseudo-curly top virus), and the potential S-acylation sites were predicted by CSS-PALM (Ren et al. 2008). Interestingly, The C4 homologs from different genera of geminiviruses (both monopartite and bipartite members) shared a conserved N-terminal motif. The result indicated cysteine residues with high scores in the S-acylation site prediction were found in these C4 proteins from different geminiviruses (Fig. 1a). Among them, the S-acylation sites on BSCTV C4 and MYMV AC4 have been verified in biochemical assays (Carluccio et al. 2018; Li et al. 2018). This analysis suggested that S-acylation may be a conserved target for geminivirus resistance in a broad spectrum. Thus, we tried to establish a strategy to improve plant resistance to geminiviruses via C4 de-S-acylation. We initiated the identification for the de-S-acylation enzyme of C4 and tested the function of this enzyme in the suppression of C4 function and geminivirus infection (Fig. 1b).

**Fig. 2:**
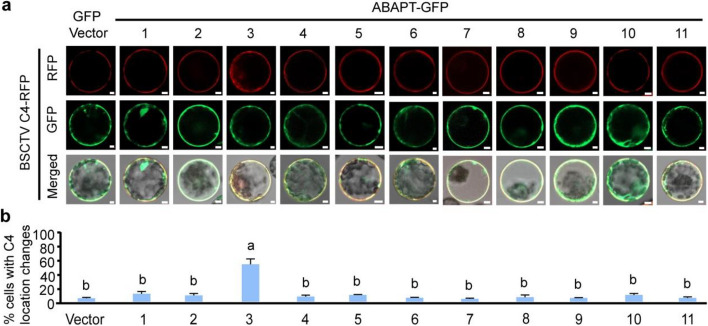
Identification of the de-S-acylation enzyme of BSCTV C4. BSCTV C4-RFP was co-overexpressed with 11 GFP-tagged ABAPT members respectively in protoplasts (GFP was used as a negative control). Cells with both RFP and GFP signals were used for analyzing the localization of BSCTV C4-RFP. The representative images of RFP, GFP, and merged signals from three biologically independent experiments are shown in (**a**). Bars, 5 µm. Percentages of cells with C4-RFP mislocalization in (**b**) are means ± SD from three biologically independent experiments (100 cells for each sample in each replicate). Significance was analyzed using one-way ANOVA followed by Tukey’s multiple comparison tests (*P* < 0.05)

Because our previous study showed that S-acylation is essential for the plasma membrane localization of C4 from BSCTV (Li et al. 2018), a geminivirus that belongs to the genus *Curtovirus*, dynamic localization changes of BSCTV C4 would be a perfect marker for fast screening of its de-S-acylation enzyme. Therefore, BSCTV C4-RFP was co-overexpressed with free GFP (as the control) or 11 GFP-fused ABAPT members respectively. As a result, the plasma membrane localization of BSCTV C4-RFP was not altered by overexpression of free GFP or most ABAPT-GFP members. Exceptionally, overexpression of ABAPT3-GFP resulted in the diffusion of BSCTV C4-RFP from the plasma membrane to the cytoplasm (Fig. 2a, b), suggesting that ABAPT3 is a de-S-acylation enzyme of BSCTV C4.

**Fig. 3:**
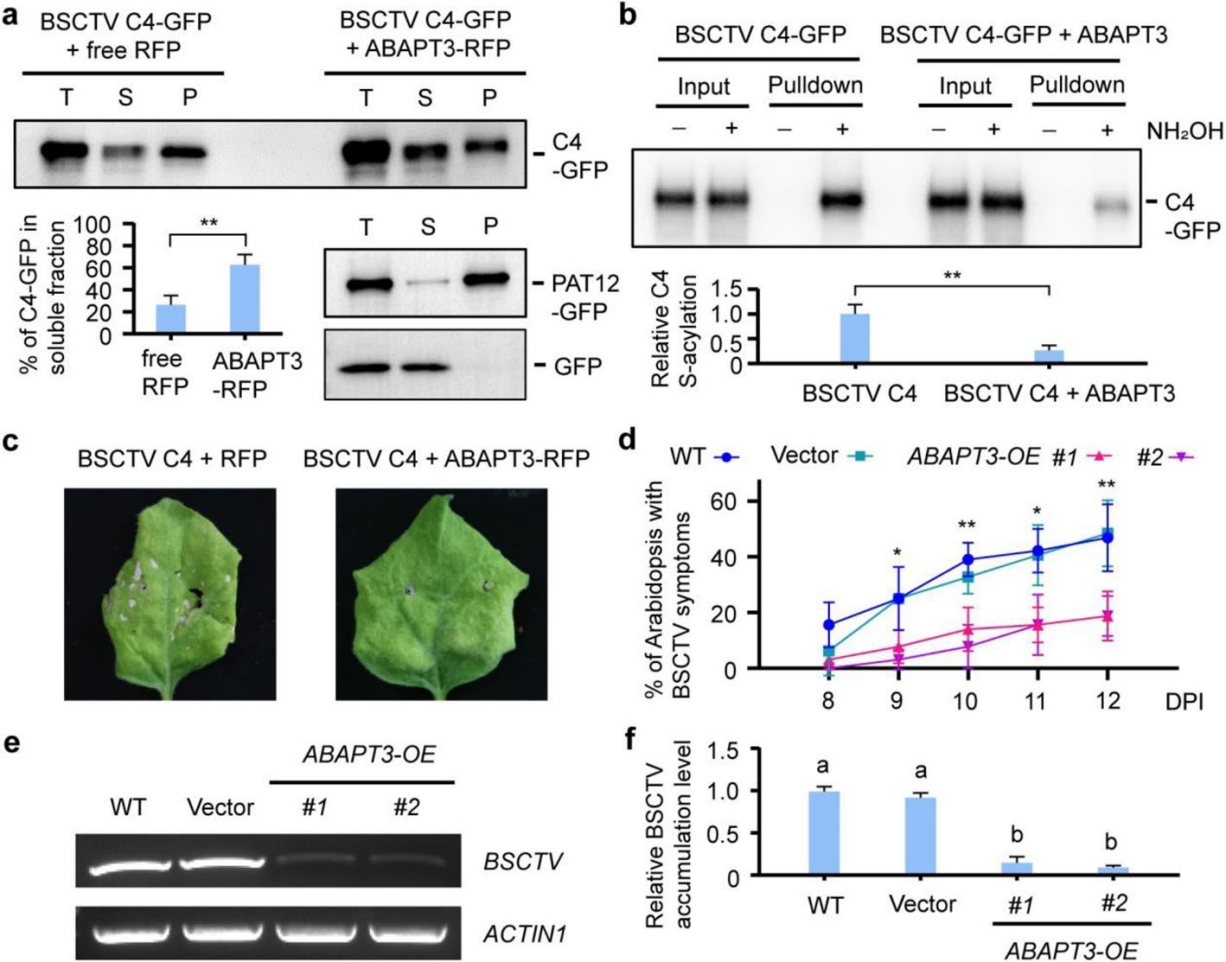
The effect of ABAPT3 overexpression on the C4 function and infection of BSCTV. **a** The effect of ABAPT3 overexpression on the membrane localization of BSCTV C4 in a cell fractionation assay. Total proteins (T) were divided into soluble (S) and pellet (P) fractions via ultra-centrifugation. The anti-GFP immunoblot is representative of three biologically independent experiments. The percentages of BSCTV C4-GFP in the soluble fraction were calculated from relative immunoblotting signals (S/[S + P]). The quantitative data are mean ± SD from three biologically independent experiments. **P < 0.01, Student’s *t*-test. The specificity of the cell fractionation assay was verified using a transmembrane protein PAT12-GFP and the free GFP as controls for pellet and soluble fractions, respectively. **b** The effect of ABAPT3 on the S-acylation of BSCTV C4. The S-acylation level of BSCTV C4-GFP with or without ABAPT3 overexpression was detected using a biotin-switch assay. The S-acylated proteins enriched on the resin which is dependent on NH_2_OH are indicated in pulldown samples. The anti-GFP immunoblot is representative of three biologically independent experiments. The immunoblot signals were quantified by ImageJ and the S-acylation levels were calculated from relative signals ([pulldown + /input +] − [pulldown − /input −]). The relative S-acylation level of BSCTV C4-GFP in the control sample was set to 1. The quantitative data are mean ± SD from three biologically independent experiments. **P < 0.01, Student’s *t*-test. **c** The influence of ABAPT3 on the BSCTV C4 induced phenotype of *N*. *benthamiana* leaves via Agrobacteria-mediated infiltration. The representative leaf phenotype 7 days after infiltration from three biologically independent experiments is shown. **d-f** The difference of BSCTV infection symptoms in the wild-type plants, vector control plants, and *ABAPT3* overexpressing transgenic plants (OE #1 and #2). The percentages of Arabidopsis plants with symptoms were calculated on different days post-inoculation (DPI). The data are means ± SD from four independent experiments. *P < 0.05, **P < 0.01, Student’s *t*-test. The levels of BSCTV in the newly emerged shoots of two-week-old Arabidopsis plants were detected by PCR. *ACTIN1* was used as an internal control. The representative PCR images are shown in (**e**). The BSCTV accumulation levels in (**f**) were shown by relative signals (*BSCTV*/*ACTIN1*). The relative virus level in the WT sample was set to 1. The quantitative data are mean ± SD from three biologically independent experiments. Significance was analyzed using one-way ANOVA followed by Tukey’s multiple comparison tests (*P* < 0.05)

The effect of ABAPT3 on the subcellular localization of BSCTV C4 was confirmed by the cell fractionation assay (Fig. 3a). To prove that the dynamic localization change of BSCTV C4 is the result of de-S-acylation, a biotin-switch assay was performed to detect the S-acylation level of BSCTV C4 with or without ABAPT3 overexpression. The biochemical data showed that BSCTV C4 was S-acylated in plant cells but its S-acylation was dramatically suppressed by ABAPT3 overexpression (Fig. 3b), providing direct evidence that ABAPT3 is the de-S-acylation enzyme of BSCTV C4. Given that C4 is the symptom determinant of BSCTV, overexpression of BSCTV C4 in *Nicotiana benthamiana* leaves resulted in cell death in the control sample, but co-overexpression of ABAPT3-RFP completely suppressed the cell death phenotype (Fig. 3c). We then inoculated BSCTV on the wild-type, GFP vector control, and two independent lines of *ABAPT3-GFP* overexpressing transgenic Arabidopsis plants. Consistently, the percentages of plants with symptoms were significantly lower in the *ABAPT3* overexpressing lines than those in wild-type and control plants (Fig. 3d). The PCR data confirmed that the BSCTV accumulation level was dramatically decreased in the Arabidopsis plants with *ABAPT3* overexpression (Fig. 3e, f). These data supported that ABAPT3 inhibits the function of BSCTV C4 and increases the plant's resistance to BSCTV.

**Fig. 4:**
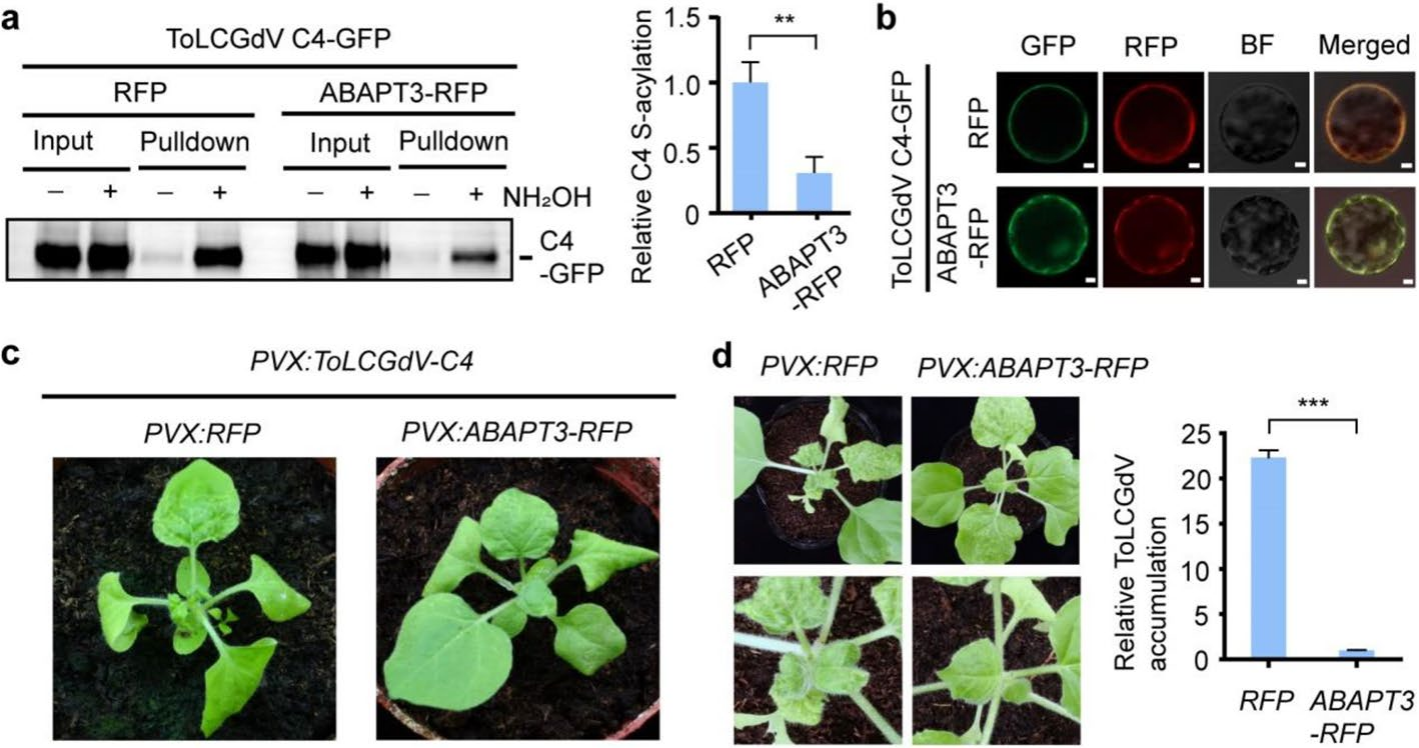
The effect of *ABAPT3* overexpression on the C4 function and infection of ToLCGdV. **a** The influence of ABAPT3 on S-acylation of ToLCGdV C4. The S-acylation level of BSCTV C4-GFP with RFP or ABAPT3-RFP overexpression was detected using a biotin-switch assay. The anti-GFP immunoblot is representative of three biologically independent experiments. The immunoblot signals were quantified by ImageJ and the S-acylation levels were calculated from relative signals ([pulldown + /input +] − [pulldown − /input −]). The relative S-acylation level of ToLCGdV C4-GFP in the RFP control sample was set to 1. The quantitative data are mean ± SD from three biologically independent experiments. **P < 0.01, Student’s *t*-test. **b** The effect of ABAPT3 on the subcellular localization of ToLCGdV C4. BF: Bright field. Bars, 5 µm. The representative images of RFP, GFP, BF, and merged signals from three biologically independent experiments are shown. **c** The influence of ABAPT3 on the function of ToLCGdV C4. *PVX:RFP* or *PVX:ABAPT3-RFP* was inoculated into *N*. *benthamiana* leaves, followed by inoculation of *PVX:ToLCGdV-C4* two days later. The phenotypes were observed 5 days after inoculation and the representative images from three biologically independent experiments are shown. **d** The effect of ABAPT3 on the symptoms of ToLCGdV infection. *PVX:RFP* or *PVX:ABAPT3-RFP* was inoculated into *N*. *benthamiana* leaves; two days later, ToLCGdV was inoculated. The symptom images recorded 12 days later are shown in the left graph. The quantitative PCR data to detect ToLCGdV accumulation shown in the right graph are means ± SD from three replications. ***P < 0.001, Student’s *t*-test

Given that the S-acylation motif is conserved in C4 from different geminiviruses, it is necessary to determine whether the de-S-acylation mediated by ABAPT3 also occurs on other geminivirus C4 proteins. Thus, we used the C4 protein from ToLCGdV, a geminivirus isolated from Guangdong province of China (belongs to the genus *Begomovirus*) (Li et al. 2020), as another example. The biotin-switch result showed that ToLCGdV C4 was also S-acylated in plant cells. Importantly, co-overexpression of ABAPT3 dramatically decreased the S-acylation level of ToLCGdV C4 (Fig. 4a), supporting that ABAPT3 also targets this geminivirus C4 protein. ToLCGdV C4-GFP was predominantly localized on the plasma membrane in protoplasts, but overexpression of ABAPT3-RFP disrupted its subcellular localization (Fig. 4b). To determine the effect of ABAPT3 on the C4 function and infection of ToLCGdV, ABAPT3-RFP was overexpressed in *N*. *benthamiana* via a Potato virus X (PVX) vector. Compared to the RFP control, ABAPT3-RFP suppressed the phenotype induced by ToLCGdV C4 (Fig. 4c). Overexpression of ABAPT3-RFP also significantly inhibited the ToLCGdV symptom and accumulation in *N*. *benthamiana*, compared to the RFP vector control (Fig. 4d). Therefore, ABAPT3 also suppresses the S-acylation of C4 to attenuate ToLCGdV infection.

In summary, based on the dynamic nature of protein S-acylation, we established a new approach to improve geminivirus resistance in plants via de-S-acylation of viral C4 proteins. Because geminivirus C4 proteins harbor a conserved S-acylation motif (Medina-Puche et al. 2021) and our results showed that overexpression of ABAPT3 inhibits S-acylation of C4 proteins from distinct geminiviruses, this method may be broadly applied to reduce infection from different types of geminiviruses. In future studies, it would be valuable to investigate whether the endogenous *ABAPT3* is upregulated under certain conditions to attenuate geminivirus infection. As overexpression of ABAPT3 does not affect plant development (Fig. S1), in our approach, ABAPT3 specifically targets the viral C4 protein and has no side effects on plant growth. Interestingly, the S-acylation of TYLCV C4, which has been reported to be distributed in multiple compartments of plant cells (Rosas-Diaz et al. 2018), was also suppressed by overexpression of ABAPT3 (Fig. S2), supporting the notion that de-S-acylation would be a general tool to regulate geminivirus C4 proteins. More importantly, this anti-virus strategy may be shared in animal and plant cells. Because many human viral proteins are targeted by S-acylation (Veit 2012), the current approach can also provide clues to prevent human virus infection. In the future, our method can be extendedly used in different plants against geminivirus infection and contribute to the improvement of disease resistance in crops.

## Materials and methods

### Plant material and growth conditions

Seeds were surface sterilized and stratified on Murashige and Skoog (MS) medium with 1.5% sucrose and 0.7% agar for 2 days in the dark and grown under a long-day condition (16/8 h of light/dark) at 22 °C for *Arabidopsis thaliana* or 26 °C for *N*. *benthamiana*. Seedlings were transferred to the soil 1 week after germination.

### Generation of constructs and transgenic plants

For co-overexpression of BSCTV C4-RFP and GFP-fused ABAPTs, *BSCTV C4* was cloned into the pCanG-*RFP* vector; the constructs for overexpression of GFP-fused ABAPTs were previously described (Liu et al. 2021). For the co-overexpression of BSCTV C4-GFP and ABAPT3, *BSCTV C4* was cloned into the pCAMBIA1302 vector, fused with *GFP*; *ABAPT3-MYC* was used to replace the *Hygromycin* (*R*) gene in pCAMBIA1302, driven under a *35S* promoter.

For overexpression of ABAPT3-RFP, the *ABAPT3* gene was cloned into the pCambia1300-*UBQ:RFP* vector. For overexpression of ABAPT3-GFP in Arabidopsis, *ABAPT3* was cloned into the pCambia1300-*35S:GFP* vector. Arabidopsis transformation was done via Agrobacterium-mediated floral dipping (Clough and Bent 1998). For the overexpression of RFP or ABAPT3-RFP in the PVX vector, *RFP* or *ABAPT3-RFP* was cloned into the *pGR106* vector. For expression of ToLCGdV C4 in protoplasts, *ToLCGdV C4* was cloned into the pCAMBIA1300-*UBQ:GFP* vector. The *PVX:ToLCGdV-C4* construct was previously described (Li et al. 2020).

### Confocal microscopy

To detect the subcellular localization in protoplasts, the constructs were transformed into leaf protoplasts (Yoo et al. 2007) from 3-week-old Arabidopsis plants. The fluorescence signals were detected under a Zeiss LSM 800 laser-scanning confocal microscope 24 h after transformation.

### Cell fractionation assay

The assay was performed as described previously (Liu et al. 2021). Protein extracts were prepared in homogenization buffer (50 mM Tris–HCl, pH 7.4, 13% sucrose, 150 mM NaCl, 1 mM EDTA, with a protease inhibitor cocktail) at 4 °C for 30 min. After centrifugation twice at 6,000* g* at 4 °C for 10 min, the supernatants were centrifuged at 80,000 g at 4 °C for 1 h to divide the extracts into soluble and pellet fractions. The pellet was resuspended in a homogenization buffer. The total lysate, pellet fraction, and soluble fraction were analyzed by SDS–PAGE and immunoblotting.

### Biotin-switch assay

The assay was performed following the previous description (Liu et al. 2021). The plasmids were transformed into protoplasts for the expression of GFP-fused proteins. after 24 h, proteins were extracted from protoplasts using lysis buffer (100 mM HEPES pH 7.5, 0.1% SDS, 1 mM EDTA, 2 mM TCEP, and 1 × protease inhibitor cocktail) and the samples were incubated at 50 °C for 5 min, then an equal volume of blocking buffer (100 mM HEPES pH 7.5, 5% SDS, 1 mM EDTA, and 0.4% MMTS) was mixed and the samples were further incubated at 40 °C for 10 min. Proteins were then precipitated by adding three volumes of acetone and incubating overnight at -20 °C, then the samples were spun at 5,000* g* for 10 min. The pellets were rinsed using 70% acetone and resuspended in 200 μL of resuspension buffer (1 × PBS pH 7.4, 2% SDS, and 8 M urea). The suspension was separated into two tubes. In each tube, the sample was incubated for 1 h with 50 μL of 4 mM biotin-HPDP, 2 μL of 100 mM EDTA, 1 μL of 100 × protease inhibitor cocktail, and supplemented with 50 μL of 2 M Tris–HCl (pH 7.4) or NH_2_OH (pH 7.4). The proteins were collected by methanol-chloroform precipitation and then dissolved in 100 μL of resuspension buffer. 20 μL of the suspension in each tube was saved as input controls, and the rest suspension was mixed with 720 μL of PBS containing 0.2% Triton X-100 and incubated with Streptavidin-Agarose for 1.5 h. The beads were then rinsed with wash buffer (1 × PBS pH 7.4, 500 mM NaCl, and 0.1% SDS) and 1 × PBS (pH 7.4). At last, 70 μL of wash buffer containing 5% β-mercaptoethanol was used to elute S-acylated proteins. The samples were detected via SDS-PAGE and immunoblotting with an anti-GFP antibody (TransGen Biotech, HT801-01).

### Virus infection

For BSCTV inoculation, 1.8 copies of the BSCTV genome in the pCambia1300 vector were used (Lai et al. 2009). The BSCTV plasmid was transformed into Agrobacterium EHA105. Agrobacteria culture was resuspended and adjusted to OD_600_ = 0.3 and used for Arabidopsis inoculation as previously described (Li et al. 2018). Symptoms with top curling were analyzed on different days after inoculation. The newly emerged shoots of the inoculated Arabidopsis plants were collected for DNA preparation for subsequent PCR to detect BSCTV DNA accumulation.

For ToLCGdV inoculation, the infectious clone pGreenII-1.3A-ToLCGdV was transformed into Agrobacterium GV3101 and inoculated on the 5–6 leaf stage *N. benthamiana* plants via infiltration as previously described (Li et al. 2020). The symptoms were observed on the indicated day after inoculation and quantitative PCR was used to detect the viral DNA accumulation.

### Supplementary Information

**Additional file 1: Fig. S1.** Verification of the *ABAPT3* overexpressing Arabidopsis plants.** Fig. S2.** The effect of ABAPT3 on the S-acylation of TYLCV C4. **Table S1.** Primers used in this study.

## Data Availability

All data generated or analyzed during this study are included in this article.

## Abbreviations

ABAPT3: Alpha/Beta Hydrolase Domain-containing Protein 17-like Acyl Protein Thioesterase 3
BSCTV: Beet severe curly top virus
ToLCGdV: Tomato leaf curl Guangdong virus
MYMV: Mungbean yellow mosaic virus
TGMV: Tomato golden mosaic virus
TYLCV: Tomato yellow leaf curl virus
TCTV: Turnip curly top virus
TPCTV: Tomato pseudo-curly top virus
PVX: Potato virus X
TCEP: Tris(2-carboxyethyl)phosphine
MMTS: S-Methyl methanethiosulfonate
Biotin-HPDP: N-[6-(biotinamido)hexyl]-3’-(2’-pyridyldithio)propionamide
DPI: Days post-inoculation
