# Supplementary material for: Improvement of plant resistance to geminiviruses via protein de-S-acylation
Source: Stress Biol. 2024 Apr 25;4(1):23. doi: 10.1007/s44154-024-00166-w (PMC11045685; doi:10.1007/s44154-024-00166-w)
Supplement: Supplementary file 1 — Additional file 1: Fig. S1. Verification of the ABAPT3 overexpressing Arabidopsis plants. Fig. S2. The effect of ABAPT3 on the S-acylation of TYLCV C4. Table S1. Primers used in this study. [file 44154_2024_166_MOESM1_ESM.pdf]

Additional file

Fig. S1 Verification of the *ABAPT3* overexpressing Arabidopsis plants

Fig. S2 The effect of ABAPT3 on the S-acylation of TYLCV C4

Table S1. Primers used in this study

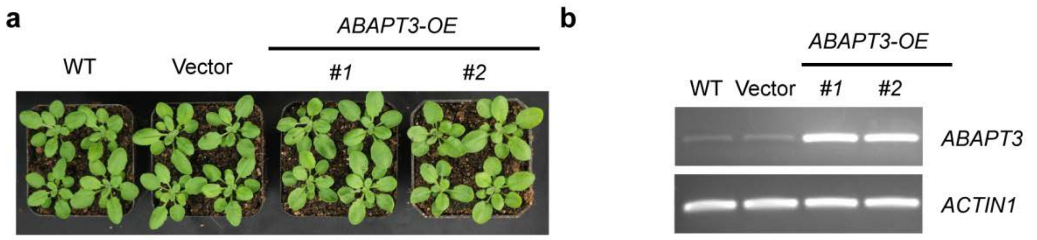

Fig. S1 Verification of the *ABAPT3* overexpressing Arabidopsis plants. **a** The phenotypes of wild-type, vector control, and *ABAPT3* overexpressing plants. The representative phenotype photograph of the 3-week-old plants is shown. **b** Verification of the level of *ABAPT3* transcripts in the *ABAPT3* overexpressing plants. RNA was prepared from the indicated Arabidopsis plants for RT-PCR. *ACTIN1* was used as an internal control. The representative data from three independent experiments are shown.

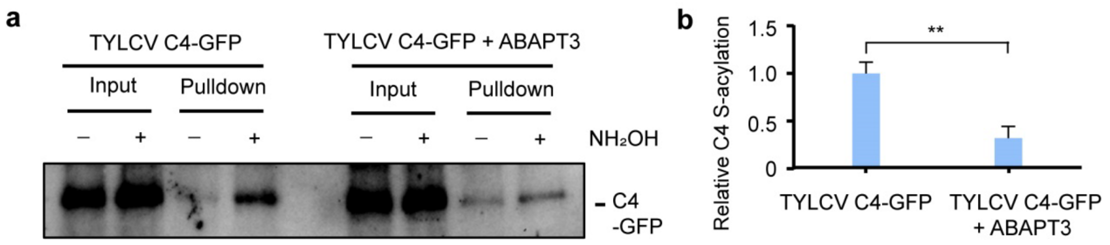

Fig. S2 The effect of ABAPT3 on the S-acylation of TYLCV C4. The S-acylation level of TYLCV C4-GFP with or without *ABAPT3* overexpression was measured in a biotin-switch assay. The S-acylated proteins enriched on the resin which is dependent on NH_2OH are indicated in pulldown samples. The representative anti-GFP immunoblot from three biologically independent experiments is shown (**a**). The immunoblot signals were quantified by ImageJ and the S-acylation levels were calculated from relative signals ([pulldown+/input+] − [pulldown−/input−]). The relative S-acylation level of TYLCV C4-GFP in the control sample was set to 1. The data in (**b**) are mean ± SD from three biologically independent experiments. $^{**}P < 0.01$, Student's *t*-test.

Table S1. Primers used in this study

Primers	Sequence (5'-3')
pCanG-35S:BSCTV-C4-RFP-F	AGTTCTAGAATGAAAATGGGGAACCACATC
pCanG-35S:BSCTV-C4-RFP-R	AGTCTCGAGAATGCCTCTGCTGCAGCATC
pCambia1302-35S:BSCTV-C4-GFP-F	ACGGGGGACTCTTGACCATGAAAATGGGGAACCACAT
pCambia1302-35S:BSCTV-C4-GFP-R	AAGTTCTTCTCCTTTACTAGTATGCCTCTGCTGCAGCATCA
pCambia1302-35S:ABAPT3-MYC-F	TACAAATCTATCTCTCTCGAGATGGGAGGTGTGACGTCATC
pCambia1302-35S:ABAPT3-MYC-R	ATTATTATGGAGAAACTCGAGTTACAGATCCTCTTCAGAGATGAGTTTCTGCTCCTCAGACCGTACTCT
UBQ:ABAPT3-RFP-F	TTTTCTGATTAACAGGGATCCATGGGAGGTGTGACG
UBQ:ABAPT3-RFP-R	AGTGGTACCCCCGGGGGATCCCTCAGACCGTACTCT
35S:ABAPT3-GFP-F	GGGGGACTCTTGACCATGGGAGGTGTGACGTCATC
35S:ABAPT3-GFP-R	CGGGCCCGCGGTACCCTCAGACCGTACTCTATCCA
BSCTV-PCR-F	AGTTCTAGAATGAAAATGGGGAACCACATCTG
BSCTV-PCR-R	AGTCTCGAGATGCCTCTGCTGCAGCATCATTA
ACTIN1-PCR-F	CTACGAGCAGGAACTCGAGA
ACTIN1-PCR-R	GATGGACCTGACTCGTCATAC
UBQ:ToLCGDV-C4-GFP-F	GTTTTTCTGATTAACAGGGATCCATGGGAACCCTCATCTCC
UBQ:ToLCGDV-C4-GFP-R	GCTCCTCGCCCTTGCTCTCGAGACTAGTAAGCCTCTGCACATGCGTCG
pGR106-ABAPT3-F	GAGGTCAGCACCAGCTAGCATCGATATGGGAGGTGTGACGTCATCA
pGR106-RFP-F	GGTCAGCACCAGCTAGCATCGATATGGCCTCCTCCGAGGAC
pGR106-RFP-R	CTATCAAGCTTATCGGCGGTCGACTTACTTGTACAGGCCG
